# In vitro validation and characterization of pulsed inhaled nitric oxide administration during early inspiration

**DOI:** 10.1007/s10877-021-00689-x

**Published:** 2021-03-18

**Authors:** Philipp A. Pickerodt, Moritz B. T. Hofferberth, Thilo Busch, Martin Russ, Mahdi Taher, Willehad Boemke, Steffen Weber-Carstens, Rainer Köbrich, Erik Swenson, Maria Deja, Roland C. E. Francis

**Affiliations:** 1 Department of Anesthesiology and Operative Intensive Care Medicine (CCM/CVK), Charité - Universitätsmedizin Berlin, Corporate Member of Freie Universität Berlin, Humboldt-Universität Zu Berlin, and Berlin Institute of Health, Campus Virchow-Klinikum, Augustenburger Platz 1, 13353 Berlin, Germany; 2 EKU Elektronik GmbH, Leiningen, Germany; 3 Division of Pulmonary, Critical Care and Sleep Medicine, University of Washington, Seattle, WA USA; 4 VA Puget Sound Health Care System, Seattle, WA USA; 5 Department of Anesthesiology and Intensive Care Medicine, University of Schleswig-Holstein, Lübeck, Germany

**Keywords:** Nitric oxide, Inhalation, PiNO, Mechanical ventilation, Artificial lung, ARDS

## Abstract

**Purpose:**

Admixture of nitric oxide (NO) to the gas inspired with mechanical ventilation can be achieved through continuous, timed, or pulsed injection of NO into the inspiratory limb. The dose and timing of NO injection govern the inspired and intrapulmonary effect site concentrations achieved with different administration modes. Here we test the effectiveness and target reliability of a new mode injecting pulsed NO boluses exclusively during early inspiration.

**Methods:**

An in vitro lung model was operated under various ventilator settings. Admixture of NO through injection into the inspiratory limb was timed either (i) selectively during early inspiration (“pulsed delivery”), or as customary, (ii) during inspiratory time or (iii) the entire respiratory cycle. Set NO target concentrations of 5–40 parts per million (ppm) were tested for agreement with the yield NO concentrations measured at various sites in the inspiratory limb, to assess the effectiveness of these NO administration modes.

**Results:**

Pulsed delivery produced inspiratory NO concentrations comparable with those of customary modes of NO administration. At low (450 ml) and ultra-low (230 ml) tidal volumes, pulsed delivery yielded better agreement of the set target (up to 40 ppm) and inspiratory NO concentrations as compared to customary modes. Pulsed delivery with NO injection close to the artificial lung yielded higher intrapulmonary NO concentrations than with NO injection close to the ventilator. The maximum inspiratory NO concentration observed in the trachea (68 ± 30 ppm) occurred with pulsed delivery at a set target of 40 ppm.

**Conclusion:**

Pulsed early inspiratory phase NO injection is as effective as continuous or non-selective admixture of NO to inspired gas and may confer improved target reliability, especially at low, lung protective tidal volumes.

## Introduction

Nitric oxide (NO), a colorless, odorless gas, is a gaseous signaling molecule exerting multiple physiological functions. Since the first description that inhalation of NO can be exploited for the treatment of pulmonary hypertension and the severe acute respiratory distress syndrome (ARDS), admixture of inhaled NO during mechanical ventilation has spread considerably: clinicians use inhaled NO to induce pulmonary vasodilation in ventilated regions of the lung which, in turn, can reduce pulmonary artery pressure and redirect blood flow towards these ventilated lung regions resulting in improved blood oxygenation [1, 2]. At concentrations of up to 80 parts per million (ppm), inhalation of NO is almost void of systemic, extrapulmonary effects, because NO is rapidly inactivated through binding to hemoglobin once it reaches the pulmonary circulation [3]. Improvements in arterial oxygenation can be achieved with concentrations ranging from as little as 0.5–20 ppm, whereas higher concentrations of up to 80 ppm of inhaled NO are typically required to reduce the pulmonary vascular resistance [4]. Despite these beneficial effects of inhaled NO on arterial oxygenation and the pulmonary circulation of ARDS patients, there is no evidence that inhalation of NO improves survival in this heterogeneous patient population. Therefore, inhaled NO is considered a rescue therapy for patients with severe ARDS and refractory life-threatening hypoxemia, including patients with COVID-19 related lung failure [5–8].

NO administration devices are commercially available to treat ventilated patients with inhaled NO. Admixture of NO to the inspired gas is brought about by continuous or flow proportional injection of NO, taken from gas cylinders of 400–1000 ppm NO in nitrogen, into the inspiratory limb of the breathing circuit at a point near the inspiratory outlet of the ventilator. NO is thereby getting diluted with oxygen and air ejected from the ventilator to the desired therapeutic concentration. For accurate dosing, the amount of NO injected has to be closely matched with the level of dilution occurring in the inspiratory limb. Therefore, the NO administration device requires intermittent manual input or direct wired information from the ventilator about the current gas flow in the inspiratory limb or the inspiratory minute ventilation. Customary NO administration devices offer two different patterns of NO injection: First, a constant rate injection of a set quantity of NO independent of the respiratory cycle. In this mode, the NO administration device requires the input of the approximate minute ventilation (i.e. the level of dilution) that is being applied to the patient. Second, a flow proportional injection of NO where NO is injected mostly during inspiration and halted when ventilator flow is zero. A flow sensor installed in the inspiratory limb of the respiratory circuit or a physical data connection between the ventilator and the NO administration device is mandatory to trigger and synchronize NO injection with inspiratory flow at each inspiration.

Independent of the mode of administration, the desired NO target concentration in the inspiratory gas must be selected (target input) and NO is then injected into the inspiratory limb of the breathing circuit close to the ventilator. Thus, mixture of NO, oxygen and air takes place within the inspiratory limb before the tidal volume reaches the patient. Side stream sampling of mixed gas is typically installed at a sampling point close to the patient to quantify and control the applied NO concentrations using a built-in electrochemical sensor.

Of importance, in vivo, during inspiration of any given tidal volume, only the gas volume inhaled at the beginning of the inspiration reaches the gas exchange area and therefore, the effect site of NO in the lung. In contrast, the gas inspired during end-inspiration fills the anatomical dead space and does not reach the alveolar space. Thus, a bolus of nitric oxide injected in synchrony with the start of inspiration and completed within no more than about the first third of the inspiration should suffice to produce therapeutic concentrations of NO at the effect site, responsible for the physiological effects of inhaled NO, and therefore be equally effective as the customary continuous and non-selective modes of administration described above. Studies in animal models and healthy volunteers, using custom-built NO administration devices support this concept. [9–12]. We hypothesize that pulsed early inspiratory phase administration of NO (“pulsed delivery”), also referred to as piNO in the literature, is feasible and equally effective in reaching the desired target concentrations. Since “pulsed delivery” restricts NO injection to the fraction of the tidal volume reaching the lung, we hypothesize that “pulsed delivery” is associated with better agreement between the set target and the yield inspiratory and intrapulmonary concentration.

Here we test the effectiveness and target reliability of a new mode injecting pulsed NO boluses exclusively during early inspiration. We (i) test if “pulsed delivery” is feasible using a commercially available NO administration device, (ii) evaluate agreement of set target and yield concentrations during “pulsed delivery” and customary administration with different injection sites, and (iii) analyze the impact of ventilator conditions on the target reliability of these NO administration modes. Further, we report intratidal peak NO concentrations and exemplify the failure of low temporal resolution electrochemical NO sensors to reflect the NO effect site concentrations during “pulsed delivery”.

## Methods

### In vitro lung model

This experimental study exclusively includes in vitro experimentation not involving animals or patients. No approval of any local or state authority or ethic commission was necessary to perform this study.

**Fig. 1:**
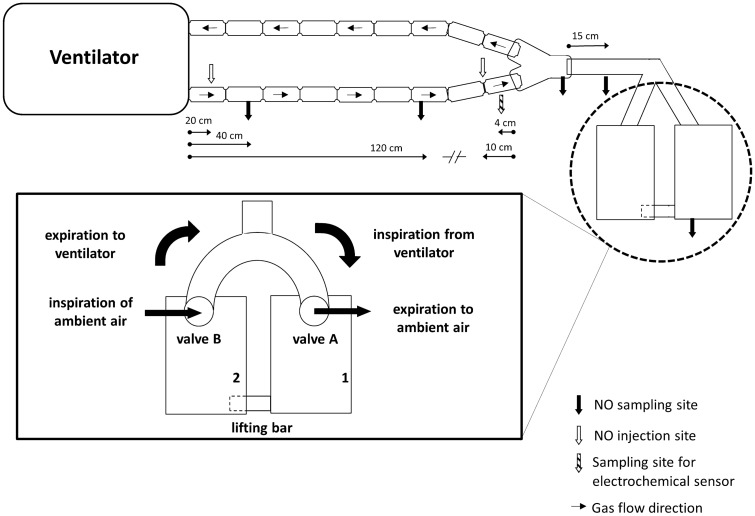
Schematic setup of the artificial lung model. Mechanical breaths from the ventilator inflate lung 1 (valve A), thereby raising a lifting bar to cause aspiration of ambient air into lung 2. Expiration into the breathing system occurs from lung 2 (valve B), while lung 1 releases to ambient air. Sites of NO injection and sampling and the distance from the ventilator or Y-piece are indicated along the breathing circuit. Injection: 20 cm distal from the ventilator, 10 cm proximal of Y-piece. Sampling: 40 and 120 cm distal from ventilator, 4 cm proximal of Y-piece, Y-piece, mid-tracheal, and artificial lung 1. Figure adapted and modified from [13]

An artificial lung model was designed and connected to a ventilator as described previously by *Imanaka* and *Kirmse* using two precision test lungs (QuickLung™, IngMar Medical, Pittsburgh, USA) [13, 14]. Both lungs were connected via a lifting bar. During inflation of lung 1 with a tidal volume containing NO, lung 2 passively fills with ambient air. During expiration, lung 1 emits the NO-spiked tidal volume to the ambient air, while lung 2 releases its NO-void tidal volume to the expiratory limb of the ventilator, thus mimicking complete uptake of NO within this artificial lung model. Two valves (Oxylog® 2000/3000, Nb. 8412001, Dräger Medical Ag & Co. KG, Lübeck, Germany) were used to direct the air flows, to separate inspiration and expiration of both lungs, and to allow “flushing” of the dead space (i.e. the apparatus dead space, artificial trachea and mainstem bronchi) with ambient air from lung 2 at each expiration [14, 15]. Figure 1 depicts the experimental set up.

An adult breathing circuit of 22 mm inner diameter (Model RT200, Fisher & Paykel Healthcare, Berkshire, UK) was used. The final length of the inspiratory and expiratory limb was 182 (ventilator to y-piece) and 160 cm (y-piece to ventilator), respectively. An endotracheal tube of 9 mm inner diameter and 30 cm length served as artificial trachea. Sites of injection of NO into and sampling from the respiratory circuit are detailed in Fig. 1. An Evita™ XL mechanical ventilator (Draegerwerk AG & Co.KG, Lübeck, Germany) equipped with software version 07.02 was used to ventilate the artificial lung model. Medical oxygen and air were rid of nitric oxides (NO_x_) and ozone (O_3_) through purification by a zero-air generator (PAG003, Eco Physics GmbH, München, Germany) before allowing inflow to the artificial lung model.

### Nitric oxide administration and quantification

A standard CARDINO™ NO administration device (EKU Elektronik GmbH, Leiningen, Germany) equipped with software version 1.4.4 was connected to the ventilator via the COM port using a protocol approved by both devices’ manufacturers. NO at a concentration of 800 ppm in N_2_ was provided from a gas cylinder (INOmax 800 ppm, INO Therapeutics AB, Lidingö, Sweden) connected to the NO administration device. Three different modes of NO administration were applied: Admixture of NO was brought about by injection of NO (800 ppm) into the inspiratory limb in the form of (i) a bolus injected in synchrony with the start of inspiration and completed within no more than the first third of the inspiration or (ii) a flow proportional injection where NO is injected mostly during inspiration and halted when ventilator flow is zero or (iii) a non-selective, continuous injection throughout the entire respiratory cycle. These administration modes resulted in either pulsed early inspiratory phase administration (piNO), referred to as “pulsed delivery” as compared to “flow proportional delivery”, and “continuous delivery”. NO was injected at 20 cm of the inspiratory limb or at 10 cm proximal from the y-piece. For the routine clinical use, the CARDINO™ is equipped with an electrochemical sensor for the quantification of NO and NO_2_ in side-stream samples drawn from the inspiratory limb at 4 cm before the y-piece and at a flow rate of 180 ml/min. With a time to detect a 90% change of the NO concentration (T90) of 10 s, and one respiratory cycle taking no more than 4 s (respiratory rate = 15/min), this sensor is to slow to detect intra-tidal changes of the NO concentration. The electrochemical sensor was calibrated according to the manufacturer’s instructions. For the purpose of accurate quantification of NO in this experimental in vitro model, a high precision, fast-responding ozone-based chemiluminescence NO analyser (NOA™ 280i, Sievers Instruments Inc., Boulder, USA; T90 = 67 ms) was used to quantify NO in samples taken at a flow rate of 200 ml/min from various sites of the lung model (Fig. 1). A two-point calibration of the ozone-based chemiluminescence was performed once daily before the experimentation with the use of an additional zero NO gas filter (ACT 01400, Sievers Instruments Inc., Boulder, USA) and a custom-made calibration gas containing 90 ppm NO in N_2_ (Nb. A 04010110, Westfalen AG, Münster, Germany).

### Measurements of inspiratory flow and airway pressures

A Fleisch-style pneumotachograph (Model No 2.7128, Metabo, Epalinges, Switzerland) was positioned in the inspiratory limb directly after the inspiratory outlet of the mechanical ventilator. The pneumotachograph was connected to a pressure transducer (ICU-Lab Pressure Box, KleisTek Advanced Electronics Systems, Bari, Italy). An identical pressure transducer was used to record the airway pressure at the Y-piece before the artificial trachea. The pneumotachograph and pressure transducers were calibrated using a 2 L calibration syringe (Model No 720253, Erich Jaeger GmbH, Höchberg, Deutschland) for flow calibration or a precision mercury manometer for pressure calibration, respectively.

### Design of experiments

Three different NO administration modes were studied at various NO target concentrations and under various ventilator conditions (Tables 2 and 3). For each individual combination of these variables, 30 consecutive respiratory cycles (respiratory rate 15/min) were recorded over a time period of 120 s. Each of these recordings was preceded by a 180 s equilibration period.

Volume-controlled ventilation (VCV; intermittent positive pressure ventilation) with rectangular gas flow and pressure-controlled ventilation (PCV; biphasic positive airway pressure ventilation) with decelerating gas flow were used. For VCV, peak inspiratory gas flow was set at 35 L/min. In order to establish a residual volume in the artificial lung model, a positive end-expiratory pressure (PEEP) of 3 cmH_2_O was set. A respiratory rate of 15/min was applied with I:E ratios of 1:1.9 and 1:1.

NO target concentrations were 5, 10, 20, and 40 ppm. The yield NO concentrations were quantified in gas samples drawn from the inspiratory limb at 40 or 120 cm distal from the mechanical ventilator, at the y-piece, at a mid-tracheal point and in artificial lung 1.

The following tidal volumes were used to emulate different ventilation strategies in a hypothetic patient of 75 kg of body weight (bw)

(i) 230 ml (approx. 3 ml/kg bw) to mimic “ultra-low” tidal volume ventilation of patients receiving extracorporeal support of gas exchange [16].
(ii) 450 ml (6 ml/kg bw) to mimic low, “lung protective” tidal volume ventilation of patients at risk of ventilator-induced lung injury [6].
(iii) 750 ml (10 ml/kg bw) to mimic traditional, “non-protective” tidal volume ventilation.

In those experiments investigating the “continuous delivery” mode of NO administration at a tidal volume of 230, 450, and 750 ml, a minute ventilation of 3.5, 6.5, and 11 L/min, respectively, was entered manually into the required input settings of the NO administration device.

The mechanical ventilator was set at an inspiratory fraction of oxygen (F_I_O_2_) of 1.0 in the intention to provoke formation of NO_2_. The safety threshold concentration of NO_2_ in the inspiratory gas mixture avoiding toxicity was defined at 2 ppm [17, 18]. Of note, we did not control for possible methemoglobinemia since all experiments were performed in vitro without blood.

### Data acquisition and statistical analysis

Airway pressures, inspiratory flow rates and the NO concentrations measured by the chemiluminescence analyser were recorded using a Powerlab™ data acquisition system (Model 8/30, ADInstruments GmbH, Spechbach, Germany) with LabChart™ 7 Pro software (version 7.3.7, ADInstruments GmbH, Spechbach, Germany). The temporal delay of detection of NO caused by the transport time of the gas through the sampling line and the processing time of the NOA™ 280i analyser was 1421 ms (“transport delay”). That temporal delay (shift) was compensated for by shifting the time axis to align data recordings. NO and NO_2_ concentrations measured by the electrochemical cell of the NO administration device were recorded manually at 60 and 120 s into the observation of each individual study condition.

**Fig. 2:**
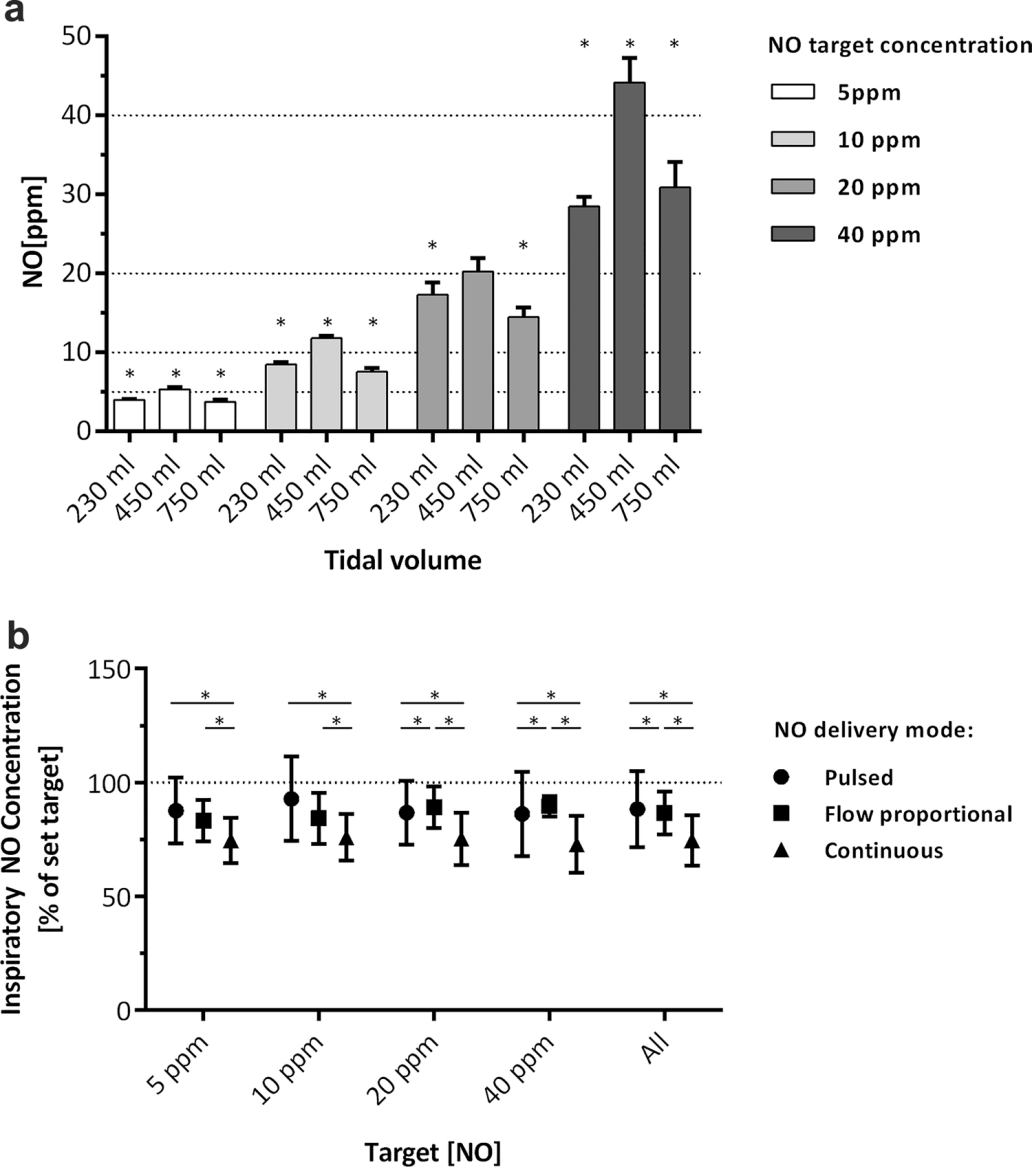
**a** Inspiratory NO concentrations achieved with “pulsed delivery”. NO was injected as a bolus during early inspiration into the breathing circuit at 20 cm after the mechanical ventilator, sampled at 120 cm thereafter, and quantified with ozone-based chemiluminescence. Ultra-low (230 ml), low (450 ml) or traditional (750 ml) tidal volumes were applied via both pressure and volume-controlled ventilation with I:E ratios of 1:1 and 1:1.9 (4 conditions). Mean NO concentrations were determined for 120 s (n = 30 respiratory cycles) per each individual ventilation condition (i.e. n = 120 per bar representing 4 ventilation conditions). Means ± SD. *p < 0.05 vs. target. **b** Target reliability of NO administration modes. NO was administered via “pulsed”, “flow proportional” or “continuous delivery” through injection into the breathing circuit at 20 cm after the mechanical ventilator, sampled at 120 cm thereafter, and quantified with ozone-based chemiluminescence. For each NO target concentration, mechanical ventilation was performed with ultra-low (230 ml), low (450 ml) or traditional (750 ml) tidal volumes applied via both pressure and volume-controlled ventilation with I:E ratios of 1:1 and 1:1.9 (12 conditions). NO concentrations were determined for 120 s (n = 30 respiratory cycles) per each individual ventilation condition (i.e. n = 360 per data point representing 12 ventilation conditions; n = 354 for “flow proportional delivery” at 5 ppm). The level of agreement is depicted by the mean inspiratory NO concentration expressed as a percentage of the target value ± SD. *p < 0.05 between respective modes

For each breath the minimum, maximum and mean NO concentrations and airway pressures, the start and duration of the inspiration, as well as the tidal volume were determined using the analysis tools of the LabChart™ 7 Pro software. In order to quantify the volume (µl) of NO gas passing a sampling site per breath, the time integral of a curve representing the mathematical product of the NO concentration and the flow rate at any given time point was calculated. The mean NO concentration per breath was calculated by dividing the volume (µl) of NO gas passing a sampling site per breath by the tidal volume measured with the pneumotachograph. Statistical analyses and production of Fig. 2a and b were performed using Prism 6 (Version 6.01, GraphPad Software, La Jolla, USA) and Excel 2013 (Microsoft Corporation, Redmond, USA). Comparisons of the mean NO concentrations per breath were performed using the Wilcoxon signed rank test. To compare the mean NO concentration in the inspiratory gas mixture and in the artificial lung model between different modes of NO administration, the Kruskal–Wallis-test using Dunn’s correction for multiple testing was used. For the comparison of NO concentrations in the artificial lung resulting from different sites of NO injection, equality of variances were checked using the Br*own-Forsythe-*test, and the *Welch’s* t-test was applied in case of unequal variances. Data are expressed as means with standard deviation (SD) unless indicated otherwise. For all statistical tests an error probability of α = 0.05 was defined. A *p*-value < 0.05 was considered significant.

## Results

### Mean inspiratory NO concentrations

To test the overall reliability of the NO administration device to reach a set inspiratory target concentration with “pulsed delivery” at ultra-low, low, and “traditional” tidal volumes, we determined the mean NO concentration in the inspiratory limb at 100 cm distal from the NO injection site achieved with various modes of ventilation (Fig. 2a). At ultra-low (230 ml) and traditional (750 ml) tidal volume ventilation (all modes combined), we found mean inspiratory concentrations consistently lower than the set target. At tidal volumes of 450 ml, however, the mean inspiratory concentrations reached 5.34 ± 0.28, 11.81 ± 0.35, 20.26 ± 1.73, and 44.19 ± 3.13 ppm, and therefore were either at or above the set targets of 5, 10, 20 and 40 ppm.

Next, we determined whether the level of agreement and/or deviation of the mean inspiratory concentration from the set target depended on the specific NO administration mode (Fig. 2b). With “pulsed”, “flow proportional”, or “continuous delivery”, the mean difference from the 5 ppm target was − 0.62 ± 0.72, − 0.83 ± 0.46, and − 1.27 ± 0.5 ppm, respectively. Similarly, the mean differences were − 0.71 ± 1.86, − 1.57 ± 1.12, and − 2.41 ± 1.03 ppm from the 10 ppm target, − 2.64 ± 2.8, − 2.16 ± 1.82, and − 4.94 ± 2.29 ppm from the 20 ppm target, and − 5.47 ± 7.42, − 4.16 ± 1.84, and − 10.9 ± 5 ppm from the 40 ppm target. All target concentrations combined, the mean inspiratory NO concentration achieved with “pulsed delivery” was 88 ± 17% of the set target, was 87 ± 9% with “flow proportional delivery”, and 75 ± 11% with “continuous delivery” (Fig. 2b).

### Mean intrapulmonary NO concentrations

Next, we determined if the yield intrapulmonary (physiological effect site) concentrations of NO, i.e. the concentration reaching the single compartment of lung 1 of the artificial lung model, was different between different modes of ventilation and NO administration. We found that the mean NO concentrations sampled from lung 1 were consistently lower than the respective set targets (Table 1), irrespective of the ventilation strategy, the NO administration mode, or the level of the target concentration. That said, at low tidal volume (450 ml), “pulsed delivery” yielded higher intrapulmonary NO concentrations (closer to target) than the other administration modes, and this result occurred consistently with all the various ventilation strategies and NO targets. Likewise, at ultra-low tidal volumes (230 ml), “pulsed delivery” yielded intrapulmonary NO concentrations closer to target, but only at the “lower” target range of 5 and 10 ppm. At the 20 and 40 ppm target, “pulsed delivery” yielded intrapulmonary NO concentrations that were similarly below target as those yielded by the other modes. At “traditional” tidal volumes (750 ml), “pulsed delivery” consistently fell below the agreement observed with the other modes of administration (Table 1).

**Table 1:** Intrapulmonary nitric oxide concentrations achieved with different modes of administration

NO target concentration	Tidal volume	Mode of ventilation	I:E	Intrapulmonary NO [ppm]		
				Delivery mode of NO administration		
	(ml)			Pulsed	Flow proportional	Continuous
5 ppm	230	PCV	1:1	3.14 ± 0.02	3.12 ± 0.02	2.38 ± 0.06**
			1:1.9	3.26 ± 0.02	2.99 ± 0.02**	3.17 ± 0.02**
		VCV	1:1	3.45 ± 0.02	3.19 ± 0.02**	3.38 ± 0.02**
			1:1.9	3.43 ± 0.03	3.17 ± 0.02**	3.4 ± 0.02*
	450	PCV	1:1	4.27 ± 0.03	3.32 ± 0.02**	3.11 ± 0.04**
			1:1.9	4.35 ± 0.04	3.34 ± 0.02**	3.46 ± 0.02**
		VCV	1:1	4.29 ± 0.02	3.61 ± 0.02**	3.29 ± 0.02**
			1:1.9	4.26 ± 0.03	3.63 ± 0.01**	3.32 ± 0.02**
	750	PCV	1:1	3.27 ± 0.07	4.08 ± 0.02**	3.87 ± 0.03**
			1:1.9	3.4 ± 0.05	4.07 ± 0.01**	4.03 ± 0.03**
		VCV	1:1	3.61 ± 0.04	3.85 ± 0.01**	3.87 ± 0.02**
			1:1.9	3.66 ± 0.03	3.86 ± 0.01**	3.85 ± 0.02**
10 ppm	230	PCV	1:1	6.83 ± 0.05	6.24 ± 0.05**	6.11 ± 0.03**
			1:1.9	6.7 ± 0.03	6.35 ± 0.03**	6.31 ± 0.03**
		VCV	1:1	7.04 ± 0.02	6.63 ± 0.03**	6.65 ± 0.02**
			1:1.9	7.07 ± 0.02	6.55 ± 0.02**	6.58 ± 0.03**
	450	PCV	1:1	9.11 ± 0.05	7.27 ± 0.04**	6.94 ± 0.04**
			1:1.9	8.94 ± 0.04	7.59 ± 0.04**	6.97 ± 0.03**
		VCV	1:1	8.75 ± 0.03	7.14 ± 0.04**	6.75 ± 0.04**
			1:1.9	8.75 ± 0.04	7.46 ± 0.04**	6.71 ± 0.03**
	750	PCV	1:1	6.63 ± 0.06	8.52 ± 0.04**	8.15 ± 0.05**
			1:1.9	6.9 ± 0.04	8.27 ± 0.05**	8.01 ± 0.07**
		VCV	1:1	7.17 ± 0.06	7.9 ± 0.04**	7.81 ± 0.02**
			1:1.9	7.27 ± 0.05	8.18 ± 0.04**	7.71 ± 0.03**
20 ppm	230	PCV	1:1	13.47 ± 0.11	12.85 ± 0.45**	13.08 ± 0.07**
			1:1.9	13.2 ± 0.1	13.26 ± 0.07	13.52 ± 0.04**
		VCV	1:1	13.81 ± 0.05	13.13 ± 0.05**	14.1 ± 0.04**
			1:1.9	13.69 ± 0.04	12.96 ± 0.04**	14.04 ± 0.04**
	450	PCV	1:1	17.1 ± 0.07	15.31 ± 0.06**	14.7 ± 0.14**
			1:1.9	16.6 ± 0.04	15.57 ± 0.07**	14.96 ± 0.06**
		VCV	1:1	16.25 ± 0.21	15.17 ± 0.08**	14.34 ± 0.06**
			1:1.9	16.14 ± 0.18	15.58 ± 0.07**	14.36 ± 0.07**
	750	PCV	1:1	12.52 ± 0.3	16.64 ± 0.04**	17.33 ± 0.08**
			1:1.9	13.01 ± 0.17	16.59 ± 0.06**	17.51 ± 0.11**
		VCV	1:1	13.54 ± 0.15	15.17 ± 0.05**	17.13 ± 0.08**
			1:1.9	13.68 ± 0.19	16.09 ± 0.09**	17.02 ± 0.05**
40 ppm	230	PCV	1:1	25.11 ± 0.16	26.98 ± 0.13**	22.68 ± 0.11**
			1:1.9	24.44 ± 0.14	26.26 ± 0.11**	23.32 ± 0.05**
		VCV	1:1	25.22 ± 0.5	26.57 ± 0.07**	23.84 ± 0.09**
			1:1.9	26.16 ± 0.11	26.67 ± 0.06**	23.75 ± 0.04**
	450	PCV	1:1	32.11 ± 0.29	31.46 ± 0.21**	27.1 ± 0.27**
			1:1.9	33.19 ± 0.19	31.58 ± 0.18**	27.95 ± 0.22**
		VCV	1:1	32.86 ± 0.23	30.14 ± 0.12**	26.2 ± 0.11**
			1:1.9	32.64 ± 0.29	30.22 ± 0.1**	26.14 ± 0.14**
	750	PCV	1:1	27.25 ± 0.51	34.17 ± 0.12**	33.3 ± 0.18**
			1:1.9	27.88 ± 0.55	33.06 ± 0.1**	33.16 ± 0.18**
		VCV	1:1	29.5 ± 0.46	30.95 ± 0.13**	32.24 ± 0.11**
			1:1.9	29.75 ± 0.32	31.92 ± 0.1**	32.21 ± 0.15**

NO was administered via “pulsed”, “flow proportional” or “continuous delivery” through injection into the breathing circuit at 20 cm after the mechanical ventilator, sampled from artificial lung 1, and quantified with ozone-based chemiluminescence. For each NO target concentration, mechanical ventilation was performed with ultra-low (230 ml), low (450 ml) or traditional (750 ml) tidal volumes applied via both pressure (PCV) and volume-controlled ventilation (VCV) with I:E ratios of 1:1 and 1:1.9 (12 conditions). Mean NO concentrations were determined for 120 s (n = 30 respiratory cycles) per each individual ventilation condition. Means ± SD); I:E, inspiration-to-expiration time ratio

**p < 0.001 vs. “pulsed delivery”

### “Pulsed NO delivery” with injection close to or distant from the y-piece

To test if the target reliability of “pulsed delivery” depended on the site where NO is injected into the inspiratory limb, we compared mean intrapulmonary NO concentrations achieved after “pulsed delivery” with injection into the inspiratory limb at either 20 cm distal form the ventilator or 10 cm proximal of the y-piece (Fig. 1), under various ventilation conditions and set NO targets (Table 2). Injection of pulsed NO close to the y-piece resulted in significantly higher mean intrapulmonary NO concentrations (clinically relevant difference) than injection close to the ventilator. Only two out of 48 experimental conditions (see Table 2: target 5 ppm, 230 and 450 ml, VCV, 1:1) did not result in a clinically relevant difference.

**Table 2:** Intrapulmonary NO concentrations upon “pulsed delivery” with injection close or distant to the Y-piece

NO target concentration	Tidal volume	Mode of ventilation	I:E	Intrapulmonary NO [ppm]		
				NO injection site		p-value
	(ml)			Close to Y	Distant to Y	Mean ± SD
5 ppm	230	PCV	1:1	4.67 ± 0.1	3.14 ± 0.02	< 0.0001
			1:1.9	4.25 ± 0.1	3.26 ± 0.02	< 0.0001
		VCV	1:1	3.24 ± 0.09	3.45 ± 0.02	< 0.0001
			1:1.9	3.95 ± 0.06	3.43 ± 0.03	< 0.0001
	450	PCV	1:1	5.04 ± 0.08	4.27 ± 0.03	< 0.0001
			1:1.9	4.94 ± 0.07	4.35 ± 0.04	< 0.0001
		VCV	1:1	4.2 ± 0.04	4.29 ± 0.02	< 0.0001
			1:1.9	4.56 ± 0.05	4.26 ± 0.03	< 0.0001
	750	PCV	1:1	5.1 ± 0.08	3.27 ± 0.07	< 0.0001
			1:1.9	4.94 ± 0.05	3.4 ± 0.05	< 0.0001
		VCV	1:1	4.58 ± 0.04	3.61 ± 0.04	< 0.0001
			1:1.9	4.72 ± 0.05	3.66 ± 0.03	< 0.0001
10 ppm	230	PCV	1:1	8.36 ± 0.31	6.83 ± 0.05	< 0.0001
			1:1.9	8.07 ± 0.17	6.7 ± 0.03	< 0.0001
		VCV	1:1	7.65 ± 0.23	7.04 ± 0.02	< 0.0001
			1:1.9	7.7 ± 0.27	7.07 ± 0.02	< 0.0001
	450	PCV	1:1	9.44 ± 0.18	9.11 ± 0.05	< 0.0001
			1:1.9	9.85 ± 0.17	8.94 ± 0.04	< 0.0001
		VCV	1:1	10 ± 0.06	8.75 ± 0.03	< 0.0001
			1:1.9	9.73 ± 0.05	8.75 ± 0.04	< 0.0001
	750	PCV	1:1	9.76 ± 0.12	6.63 ± 0.06	< 0.0001
			1:1.9	9.89 ± 0.09	6.9 ± 0.04	< 0.0001
		VCV	1:1	10.24 ± 0.1	7.17 ± 0.06	< 0.0001
			1:1.9	9.8 ± 0.12	7.27 ± 0.05	< 0.0001
20 ppm	230	PCV	1:1	16.03 ± 0.67	13.47 ± 0.11	< 0.0001
			1:1.9	13.95 ± 0.4	13.2 ± 0.1	< 0.0001
		VCV	1:1	14.99 ± 0.11	13.81 ± 0.05	< 0.0001
			1:1.9	14.78 ± 0.4	13.69 ± 0.04	< 0.0001
	450	PCV	1:1	19.35 ± 0.18	17.1 ± 0.07	< 0.0001
			1:1.9	19.58 ± 0.31	16.6 ± 0.04	< 0.0001
		VCV	1:1	19.03 ± 0.12	16.25 ± 0.21	< 0.0001
			1:1.9	19.1 ± 0.1	16.14 ± 0.18	< 0.0001
	750	PCV	1:1	20.39 ± 0.28	12.52 ± 0.3	< 0.0001
			1:1.9	19.9 ± 0.16	13.01 ± 0.17	< 0.0001
		VCV	1:1	20.33 ± 0.24	13.54 ± 0.15	< 0.0001
			1:1.9	20.11 ± 0.37	13.68 ± 0.19	< 0.0001
40 ppm	230	PCV	1:1	35.2 ± 1.5	25.11 ± 0.16	< 0.0001
			1:1.9	31.86 ± 0.75	24.44 ± 0.14	< 0.0001
		VCV	1:1	32.45 ± 0.88	25.22 ± 0.5	< 0.0001
			1:1.9	33.91 ± 0.89	26.16 ± 0.11	< 0.0001
	450	PCV	1:1	39.27 ± 0.59	32.11 ± 0.29	< 0.0001
			1:1.9	39.19 ± 0.45	33.19 ± 0.19	< 0.0001
		VCV	1:1	39.14 ± 0.2	32.86 ± 0.23	< 0.0001
			1:1.9	39.04 ± 0.15	32.64 ± 0.29	< 0.0001
	750	PCV	1:1	42.15 ± 0.57	27.25 ± 0.51	< 0.0001
			1:1.9	41.14 ± 0.47	27.88 ± 0.55	< 0.0001
		VCV	1:1	40.93 ± 0.63	29.5 ± 0.46	< 0.0001
			1:1.9	40.87 ± 0.43	29.75 ± 0.32	< 0.0001

NO was injected as a bolus during early inspiration into the breathing circuit at either 20 cm after the mechanical ventilator (distant to Y-piece) or at 10 cm proximal of the y-piece (close to Y-piece), sampled from artificial lung 1, and quantified with ozone-based chemiluminescence. For each NO target concentration, mechanical ventilation was performed with ultra-low (230 ml), low (450 ml) or traditional (750 ml) tidal volumes applied via both pressure (PCV) and volume-controlled ventilation (VCV) with I:E ratios of 1:1 and 1:1.9 (12 conditions). Mean NO concentrations were determined for 120 s (n = 30 respiratory cycles) per each individual ventilation condition. Means ± SD; I:E, inspiration-to-expiration time ratio

### Intratidal peak concentrations of NO and NO_2_

Injecting boluses of NO into the breathing circuit, while limiting the NO injection to early inspiration may result in excessively high peak concentrations of NO in the inspiratory limb before dilution with the tidal motion of gas can occur along the circuit. Therefore, we determined the peak intratidal NO concentrations occurring at different sampling sites along the circuit during “pulsed delivery” with injection at 20 cm distal from the ventilator and a set target concentration of 40 ppm under one single ventilation condition (VCV, tidal volume 750 ml, I:E 1:1). Of note, peak intratidal concentrations in the inspiratory limb were manifold higher than the set target concentration, while the peak concentration ejected from the single compartment lung 1 was close to target (Table 3). Moreover, in gas samples from the y-piece, electrochemical quantification of NO (28 ppm) failed to capture the intratidal peak (103 ppm) detected with chemiluminescence (Table 3).

**Table 3:** Peak intratidal NO concentrations during “pulsed delivery” with 40 ppm target

	40 cm distal to ventilator	120 cm distal to ventilator	Y-piece	Trachea	Artificial lung
OBC	184.73	133.03	103.33	107.5	33.68
ECS	n/d	n/d	28.3	n/d	n/d

In “pulsed delivery” mode with a target of 40 ppm, a bolus of NO was injected at the beginning of inspiration into the breathing circuit at 20 cm after the mechanical ventilator, sampled from the indicated sites along the breathing circuit, and quantified with ozone-based chemiluminescence (OBC). Mechanical ventilation was performed with 750 ml of tidal volume via volume-controlled ventilation with I:E ratio of 1:1. (1 condition). Peak intratidal NO concentrations were determined over a period of 120 s (n = 30 respiratory cycles) at each sampling site. Gas samples form the y-piece also underwent quantification with the built-in electrochemical sensor (ECS at 4 cm proximal of the y-piece) of the NO administration device

*n*/*d* not determined

Finally, we determined the peak NO concentrations occurring in the artificial trachea when different set targets and modes of NO administration were used (Table 4). The tracheal peak NO concentration were highest during “pulsed delivery”, and reached peaks of 68 ± 30 and 148 ± 38 ppm when 40 ppm were targeted with NO injection distant or close to the y-piece, respectively. Upon injection of NO at the customary injection site at 20 cm distal of the ventilator, the peak NO_2_ concentrations (electrochemical sensor) occurred when the administration modes were set at a target of 40 ppm NO, and amounted to 1.0, 1.4, and 1.9 ppm NO_2_ during “pulsed”, “flow proportional”, and “continuous delivery”, respectively.

**Table 4:** Peak intratidal NO concentrations in the artificial trachea resulting from different modes and sites of NO administration

Delivery mode	Flow proportional	Continuous	Pulsed	
	Injection site relative to y-piece			
Target [NO]	Distant	Distant	Distant	Close
5 ppm	6.27 ± 1.22	8.1 ± 2.59	8.73 ± 3.41	17.83 ± 5.66
10 ppm	12.22 ± 2.58	14.81 ± 4.62	16.15 ± 5.64	29.4 ± 6.27
20 ppm	23 ± 3.69	31.02 ± 9.92	33.35 ± 13.45	73.11 ± 24.25
40 ppm	45.21 ± 7.1	59.57 ± 20	68.4 ± 30.14	147.73 ± 38.1

NO was administered via “flow proportional” or “continuous delivery” through injection into the breathing circuit at 20 cm after the mechanical ventilator (distant to y-piece), or via pulsed delivery with injection distant or close to (i.e. 10 cm proximal of) the y-piece, sampled from a mid-tracheal sampling site, and quantified with ozone-based chemiluminescence. For each NO target concentration, mechanical ventilation was performed with ultra-low (230 ml), low (450 ml) or traditional (750 ml) tidal volumes applied via both pressure (PCV) and volume-controlled ventilation (VCV) with I:E ratios of 1:1 and 1:1.9 (12 conditions). Peak intratidal NO concentrations in the trachea were determined over a period of 120 s (n = 30 respiratory cycles) per each individual ventilation condition (i.e. n = 360 per tabular cell representing 12 ventilation conditions). Means ± SD

## Discussion

We designed an in vitro lung model to test the effectiveness and target reliability of a new mode of administration of inhaled NO injecting pulsed NO boluses exclusively during early inspiration. We found that such “pulsed delivery” yielded inspiratory and intrapulmonary NO concentrations with better agreement to the set target concentration (5–40 ppm range) than customary modes of NO administration operating with admixture of NO proportional to inspiratory flow or continuously throughout the entire respiratory cycle. The target reliability of “pulsed delivery” was more evident when ultra-low and low tidal volumes were used, or when NO was injected close to the y-piece. “Pulsed delivery” produced intratidal peak concentrations of NO in the inspiratory limb manifold higher than the respective target concentration, but not detectable with electrochemical quantification. Within the 40 ppm NO target range, neither “pulsed delivery” nor any of the other NO administration modes produced NO_2_ levels above the toxic threshold of 2 ppm in the inspiratory limb.

The concept of “pulsed delivery” limiting the injection of NO into the inspiratory limb to early inspiration, relies on the notion that, in vivo, during inspiration of any given tidal volume, the volume inspired during end-inspiration does not reach the physiological effect site of NO, i.e. the alveolar gas exchange area. Agreement of the yield inspiratory and set NO concentrations achieved with “pulsed delivery” was 88% as compared to 75% with flow-independent “continuous delivery” (Fig. 2b). The target reliability of “pulsed delivery” was superior to the other modes, especially, at ultra-low (230 ml) and low (450 ml) tidal volumes, but was inferior to the other modes when a large tidal volume (750 ml) was used. During VCV with rectangular inspiratory gas flow, the time of inspiratory flow required to produce a low tidal volume is shorter than to produce a larger tidal volume, and therefore the inspiratory flow for a low tidal volume stops at an earlier point in the inspiratory phase. As a result, for low tidal volumes, a greater part of the time of inspiratory flow overlaps with the early inspiratory phase, i.e. when the pulsed NO bolus is applied, as compared to larger tidal volumes where the time of inspiratory flow extends towards the end of the entire inspiratory time. The pulsed NO bolus therefore hits more of the inspiratory flow time of a low tidal volume than of the inspiratory flow time of a larger tidal volume, and that seems to contribute to the improved target reliability of “pulsed delivery” with low tidal volumes.

Following this line of reasoning, “pulsed delivery” of NO during early inspiration may be advantageous when inhaled NO is used for patients receiving low, lung protective tidal volumes, i.e. the current gold standard of mechanical ventilation for patients with lung injuries [3–5]. Furthermore, administration of inhaled NO via non-synchronized injection of NO into the inspiratory limb, i.e. continuously throughout the entire respiratory cycle (“continuous delivery”), may put the patient at risk of receiving a significantly lower than the intended concentration and set target of inhaled NO. This is of importance and relevance for the clinical management of patients, since a variety of mechanical ventilators do not offer synchronized “flow proportional delivery” of NO administration, requiring a flow signal or physical data connection between the ventilator and the NO administration device to synchronize NO injection and inspiratory flow. Especially during intra- and interhospital transfers of ventilated patients receiving inhaled NO, flow-independent continuous NO delivery modes are often being used, due to the technical ease of use of these “continuous delivery” modes.

Hence, the switching from one administration mode to the other, may cause inadvertent administration of a lower than the targeted concentration of inhaled NO. A sudden, inadvertent decrease of the concentration of inhaled NO (upon mode switch, e.g. during transfer), can severely impair blood oxygenation or cause increases of pulmonary vascular resistance and right ventricular afterload. Having said that, it is important to note that the correct dosage of inhaled NO administered to patients, is to be titrated to the desired clinical effects, rather than to a fixed target concentration. The level of blood oxygenation and pulmonary vascular resistance are therefore important clinical target outcomes of inhaled NO.

In this artificial lung model, “pulsed delivery” injecting NO at the customary injection site at 20 cm distal of the ventilator, and therefore relatively distant from the patient, resulted in intrapulmonary NO concentrations similar to those achieved with the two other administration modes. However, “pulsed delivery” with injection close to the y-piece, and therefore relatively close to the patient, resulted in tracheal NO concentrations closer to the set target. This observation can be explained by the mixing of the pulsed NO bolus during the transport time and tidal motion of the gas along the inspiratory limb causing the “spreading” and dilution of NO beyond early inspiration. Such redistribution of the NO bolus within the applied tidal volume spreads the amount of NO delivered to the lung, partially mitigating the target reliability of “pulsed delivery” when not injected close to the patient. Of note, in many current ventilator models (except e.g. the Evita™ XL mechanical ventilator of the present study) a bias flow present in the inspiratory limb of the breathing circuit during expiration, does also contribute to spreading and dilution. The physiological importance of the timing of the NO bolus in relation to the inspiratory flow is also evident in studies of Heinonen et al*.* on spontaneously breathing horses undergoing general anaesthesia [11]. These animals received pulsed delivery of a given concentration of NO into the endotracheal tube either during the first half (30% or 60%) or the second half (50–80%) of the inspiration. Administration of NO during the first 30% and 60% significantly reduced venous admixture and thus increased the arterial partial pressure of oxygenation (P_a_O_2_). Pulsed NO delivery exclusively during the second half (50–80%) of the inspiration did neither reduce venous admixture nor increase P_a_O_2_, and therefore did not seem to have reached the alveolar physiological effect site of NO. Consequently, any time lag between the start of the inspiratory gas flow and the start of NO injection, i.e. the timing of the NO pulse, can alter the physiological effects of pulsed early inspiratory NO administration. Time lag may result from signal processing within the mechanical ventilator and the NO administration device. In addition, variable length and compliance of the tubing used to connect the NO device to the breathing circuit can contribute to lag time of the NO bolus in relation to the inspiratory flow. Consequently, a fine adjustment to harmonize the interplay between individual mechanical ventilators and NO administration devices is crucial to ensure satisfactory target reliability.

**Fig. 3:**
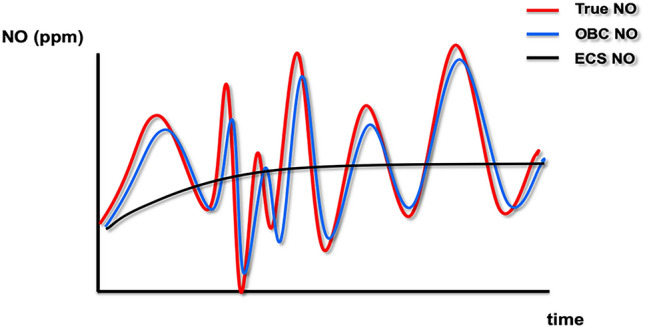
Sensitivity of NO quantification methods. The graph depicts the theoretical temporal resolution of ozone-based chemiluminescence (OBC, blue) versus an electrochemical sensor (ECS, black) in retracing fluctuations of the true NO concentration (red)

With respect to the potential need to evaluate the target reliability at the bedside, it is of relevance that the yield NO concentrations in our model remained within a range of at least 75% (“continuous delivery”) of the set target. Larger discrepancies occurred between the set target and the results of the electrochemical quantification of the yield NO concentrations displayed on the screen of the NO administration device. These differences are due to the slow response time of the electrochemical cell to changes in nitric oxide concentrations. Furthermore, common NO administration devices display the moving average of NO concentrations measured over the past 20 s, or similar. (Fig. 3). These intricacies significantly impair the usefulness of electrochemical quantification to accurately reflect intratidal peak and mean NO concentrations. This situation can cause confusion of clinicians at the bedside and bring them to put the device’s reliability into question.

Our findings have several limitations: Foremost, this is an in vitro study, investigating technical implications and feasibility, without verification of physiological effects of “pulsed delivery” in vivo. Whilst there is little reason to assume alteration of the physiological effects of inhaled NO once the desired effect site concentrations are reached, “pulsed delivery” in vivo must be applied with caution and care to verify its clinical feasibility and effectiveness. The dose–response-relationship of inhaled NO in humans has been studied elsewhere [2, 19]. Second, this study is limited to controlled modes of ventilation, without assisted or spontaneous ventilation, where high fluctuations of tidal volumes and gas flows can occur to make “pulsed delivery” even more challenging. The continuous bias gas flow of modern mechanical ventilators to detect spontaneous breathing efforts may additionally influence the distribution and dilution of the NO bolus in the inspiratory limb, when NO is injected close to the ventilator. Therefore, “pulsed delivery” was designed for injection close to the patient. Third, only one type of ventilator was used in this study, and the results cannot be extended to other combinations of technical equipment.
